# Mobility of Erasmus+ students in Europe: Geolocated individual and aggregate mobility flows from 2014 to 2022

**DOI:** 10.1038/s41597-025-04789-0

**Published:** 2025-03-24

**Authors:** Tuomas Väisänen, Milad Malekzadeh, Oula Inkeröinen, Olle Järv

**Affiliations:** 1 Digital Geography Lab, Department of Geosciences and Geography, University of Helsinki, Helsinki, Finland; 2 Helsinki Institute of Sustainability Science, University of Helsinki, Helsinki, Finland; 3 Helsinki Institute of Urban and Regional Studies, University of Helsinki, Helsinki, Finland; 4 Helsinki Inequality Initiative, University of Helsinki, Helsinki, Finland

**Keywords:** Geography, Education, Society, Research data

## Abstract

Student mobility is a distinct form of human movement. It can indicate the characteristics and attractiveness of regions, which is relevant for governance, policy, and planning. In Europe, the Erasmus+ programme has facilitated the mobility of over two million students between 2014 and 2022, and this individual-level mobility data is openly available. However, the lack of spatial information hinders its use in geographical research. In this article, we present enriched student mobility data by adding spatial information at the Local Administrative Unit (LAU) and Nomenclature of Territorial Units for Statistics (NUTS) 3 regional levels. Using the Photon geocoding service, we converted textual origin and destination locations into spatial data, creating a precise annual-level mobility dataset. The geolocated student mobility dataset contains both individual- and aggregate-level mobility flows between LAU and NUTS 3 spatial units across Europe from 2014 to 2022. We validated the geolocated data through random sampling and manual verification, achieving accuracy scores above 96%. Finally, we provide use cases for the data.

## Background & Summary

Mobilities of people for reasons such as permanent migration, daily commuting, and tourism reveal how locations and regions are connected, how societies function, and how their spatial structures and temporal dynamics change over time. Consequently, the mobility of people can be seen as a tool with which to understand society and provide new insights^1^. For example, cross-border mobility can be used to identify cross-border social interactions and how the mobility forms functional border regions^2^. Mobilities of people in cities showed how the COVID-19 pandemic changed urban mobility structures and socio-economic inequalities^3–5^. Not the least, mobility of people helps optimise public and private sector services, and provide crucial information for smart city initiatives^6^. Understanding mobilities is thus crucial for governance, policies, and planning at the local, regional and national levels.

Student mobility is a form of mobility that can be seen as a relevant indicator of the attractiveness and performance of localities from the economic and innovation perspectives. The mobility of students is one of the cornerstones in the dissemination of academic knowledge and fostering international networks of expertise^7–10^. Students going abroad not only study, but also gain personal development through interaction with other cultures, while learning languages that foster social inclusion and familiarity with diversity^9,11–13^. Students also act as consumers in the cultural, leisure, and tourism sectors of their destination countries^7,10,14^. Moreover, international student mobility is not driven by differences in wages or affordable housing as much as they affect migration and cross-border commuting. Instead, it is driven more by academic pursuits, ensuring a better salary after finishing studying, and learning about other cultures and languages^7,10^. Finally, one of the key influences on where students decide to go is the bilateral agreements between institutions^15^.

From a regional perspective, the student mobility dynamics can lead to some regions becoming “brain gain” regions by attracting more (talented) exchange students than other regions. Vice versa, “brain drain” regions are potentially losing talented future generations^16–18^. Hence, student mobility flows also contribute to producing regional hierarchies in Europe by generating attractive brain gain and stagnating brain drain regions. The production of such regions is likely to reinforce socio-economic circumstances that cause regional inequality and left-behindedness^7,18–20^. Consequently, understanding the role of student mobility for regional development and economies can provide stakeholders with new insights for planning and policies.

The European Union’s Erasmus+ programme is one of the largest student mobility networks between higher education institutions (HEIs) in the world. It has supported the mobility of nearly 16 million students since its inception in 1987^21^, and keeps playing a substantial role in fostering social cohesion of the EU and supporting the creation of a European identity for students in the EU member, partner, and candidate nations^8,11,22,23^, which also is a long-term goal of the programme^24,25^. The Erasmus+ programme is not only limited to student exchange, but it also supports exchanges of teachers and other HEI staff^8^. After the COVID-19 pandemic, the Erasmus+ programme had two of its most successful years in 2022 and 2023 to date, with over 1.2 million participants per year^21^.

Therefore, the Erasmus+ programme dataset, that is openly available and anonymized at the individual level, is a perfect source to study student mobility in Europe at large^26^. However, the data are not available in a spatial format. That is, geographical information about the origins and destinations of all student movements are in a textual format and written in various languages. This poses a challenge for using the data to study student mobility in geographical and regional research.

In this paper, we present the spatially enriched version of Erasmus+ student mobility data at LAU (Local Area Unit) and NUTS 3 (Nomenclature for Statistical Territorial Unit) spatial levels, from 2014 to 2022. The data are based on the open student mobility statistics provided by the European Union^26^. To enrich the data spatially, we used the free and open-source Photon geocoding service^27^ to geocode the origins and destinations of the student mobility for all student exchanges that lasted at least three months, and when the student was 18 years old or older.

Similar Erasmus student mobility data have previously been converted into spatial datasets by Gadár *et al*.^28^ and Breznik & Skrbinjek^8^. However, the datasets are already ten years old as they cover an earlier timeframe (2008 until 2014), and there have been no spatially enriched datasets since. On top of covering more recent years, our spatially enriched dataset covers the COVID-19 period, making this dataset highly valuable for studying potential short- and long-term changes in the spatial structures of student mobility in Europe. Furthermore, as the Erasmus+ programme’s first cycle started in 2014 and ended in 2020, and the second cycle started in 2021, it marks an excellent occasion to extend the temporal coverage of existing Erasmus+ student mobility data to cover the full first cycle of the Erasmus+ programme and the first few years of the second cycle, which ends in 2027.

Finally, for the future research, our spatially enriched student mobility data in Europe based on the Erasmus+ programme data could be further complemented by student mobility data from other student exchange initiatives, such as Fulbright scholarships, ISEP exchanges, Nordplus exchanges, institution-specific scholarships, and bilateral student exchange programmes. Such work would expand the coverage of student mobility data beyond Europe to a global level.

### Description of original data

The original data we used as input for producing this geocoded data product is available on an annual level from Directorate-General for Education, Youth, Sport and Culture^26^ with a CC BY 4.0 licence^29,30^ and at the following link: http://data.europa.eu/88u/dataset/erasmus-mobility-raw-data.

The original data contains information on student exchange movements performed under the Erasmus+ programme across Europe on a participant-level annually from 2014 to 2022. The variables in the data describe each movement in detail, and each data row represents one movement of a student. Most importantly, the data includes textual information about the sending and receiving institutions, cities, and countries. In addition to origin and destination information, the data describes the duration, starting month, gender, age, type of mobility, participant profile, ISCED (International Standard Classification of Education) study level, field of studies, and whether they have a less-privileged background, among other tertiary information (see Table 1).

**Table 1:** Variables of the original, unmodified ERASMUS+ data with their official descriptions from the Erasmus Data Dictionary^26^.

Variable	Description	Value unit
Project reference	Erasmus+ project code	Text
Academic year	Year of the mobility	Text
Mobility start	Start of the mobility	Date
Mobility duration	Duration of mobility in calendar days	Integer
Activity	Type of mobility within Erasmus+ programme	Text
Field	Field name of the action	Text
Field of Education	Field of education	Text
Participant country	Country of the participant	Text
Educational level	ISCED code representing level of education of the participant	ISCED 3-9
Participant gender	Gender of the participant	Male/female/undefined
Participant profile	Describes whether participant is a learner or staff member	Learner/staff
Fewer opportunities	Describes whether participant comes from less privileged background	Yes/No
Participant age	Age of participant	Integer
Sending country	Country of origin for participant	Text
Sending city	City of origin for participant	Text
Sending organization	Institution of origin for participant	Text
Receiving country	Country of destination for participant	Text
Receiving city	City of destination for participant	Text
Receiving organization	Institution of destination for participant	Text
Actual participants	Number of participants with identical mobility	Integer

Some variables in Table 1 representing the original variables in the Erasmus+ data require clarification. The “Field” describes the educational level a participant was partaking student mobility was enrolled at (higher education, vocational education, school education, or youth mobility). The “Field of Education” is a manually inserted description of the participant’s field of education (e.g., engineering, law, or business studies) by an institution representative. Given information filled manually, the variable contains 337 unique values, in total. The “Educational level” variable describes the ISCED levels ranging between 3 and 9 [^31^, p.71–72]. It ranges from upper secondary education (ISCED level 3) to doctoral education (ISCED level 8), and the ISCED 9 level represents a non-classified education level.

Most of Erasmus+ data contains mobilities related to countries that are European Union member states, but other countries are also included^32^. Such countries are the European Free Trade Association and European Economic Area countries (e.g. Norway, Switzerland, Iceland, Liechtenstein), EU candidate countries, such as Turkey and North Macedonia, and a few third countries outside Europe. Furthermore, the UK exited the European Union and consequently the Erasmus+ programme in 2020 with Brexit. However, while the UK has not been participating in the Erasmus+ programme since 2020, several Erasmus+ projects were funded before or in 2020, and these projects extend to 2021 and 2022, explaining the presence of post-Brexit Erasmus+ mobility to and from the UK^32,33^.

The data were provided by the EU annually as separate Excel files representing calendar years, which we combined. Given that the data include all exchange movements under the programme (e.g., staff and teachers), we first filtered the data to contain only the students we were interested in and who moved within Europe.

### Data filtering

For the spatially enriched student exchange dataset, we selected the records from the initial dataset that describe mobility of students (learners) who are at least 18 years old, and the duration of their mobility is at least 90 days. This ensures the mobility data captures mobility behaviour that is analogous to student exchanges and research visits between HEIs, and does not contain short-length trips, excursions or staff mobility. The pre-geocoded data contains mobilities of 2 372 505 individual students, who have created 2 282 950 student exchange movements between origin and destination institutions between 2014–2022. The number of individuals is higher than the number of movements, as there are a few cases when more than one person has an identical mobility between two institutions. Table 2 shows how these numbers are distributed across the years. These movements can also originate from or end in areas that are outside geographically defined Europe yet being administratively part of European Union (e.g. French overseas territories, Greenland). By excluding these student movements outside geographically defined Europe, the total numbers of mobile students reduces in our data after geocoding and spatial aggregation to the NUTS 3 and LAU spatial levels (see Section 3 for final numbers).

**Table 2:** Counts of Erasmus+ flows and students from 2014 to 2022 before geocoding.

Year	Total movements	Total participants
2014	159 980	166 071
2015	265 453	275 693
2016	281 813	293 142
2017	293 358	304 374
2018	303 223	314 974
2019	302 410	314 643
2020	159 099	165 263
2021	244 735	254 493
2022	272 879	284 032
**Total**	2 372 505	2 282 950

## Methods

### Geocoding

Geocoding is the process by which a toponym (textual place name) or a description of a place is assigned a locational reference, usually geographic coordinates^34,35^. It is widely used in online map services and journey planners to transform addresses into geographical coordinates, but also in research to transform data into spatial data^34,36,37^. The common approach in geocoding is to provide an input toponym for the geocoder, which then attempts to find a match for the toponym from one or more gazetteers^34^, however other approaches also exist^38,39^. If a match is found from the gazetteer, the geocoder returns the matching record in the form of geographic coordinates or a point-of-interest object, which refers to a specific geographical point on the surface of earth. Geocoding can also be done in reverse, whereby a coordinate point is matched with the nearest known toponym in the gazetteer.

We used the free and open-source Photon and Nominatim geocoding services implemented in the geopy Python library^40^ to geocode the toponyms in the Erasmus+ data for three reasons. First, they are free and open-source geocoding tools, and using them supports open science practices and the transparency of this work^41,42^. Second, while Photon is a based on the Nominatim geocoder by^43^, Photon has a better support for multilingual (English, German, French and local) placenames and is more tolerant of typographical errors through the use of Elasticsearch in the backend^27^. This is crucial for the Erasmus data, as toponyms in the data are written in various European languages, scripts, and can contain typographical errors. Third, both Photon and Nominatim have been found to perform well in a wide variety of geocoding tasks in previous research^36,44–47^.

While most of our geocoding is based on Photon, we also used Nominatim to support resolving toponyms that were not resolved in the first pass with Photon on original toponyms, nor on the second pass with Photon on manually corrected toponyms (roughly 5% of all toponyms). For a more detailed description of the geocoding workflow, see Section 2.1.2). As both geocoders are based on Nominatim, its place ranking backend will favour more prominent placenames in the cases of identical placenames from the same country^48^.

#### Preprocessing

The reporting of the toponyms in the Erasmus student exchange data is somewhat inconsistent, and thus geocoding the data required additional preprocessing. The country names are reported in a standardised manner, as they’re all identical for individual countries. However, the names of cities and institutions are not standardised and are manually entered into the data by the person filling the mobility entry, which leads to inconsistent toponyms for the same locations. For instance, there are several cases in which either the name of the city or the institution contains a typographic mistake, such as “Llllondon, United Kingdom”. In other cases the toponym is written in a national language (e.g., “Estocolmo” instead of “Stockholm”), or using a different script (such as Greek or Cyrillic). Furthermore, some entries have been written using various character encodings, which leads to characters with umlaut or diacritical marks to be presented erroneously with the UTF-8 symbol for a character out of range, or a plain question mark substituting for the missing character. For instance, the toponym of “Gödöllö, Hungary”, can look like “G?d?ll?, Hungary”. Finally, some entries do not report the city at all, but instead, refer to the institution through an identification number. These inconsistencies pose challenges for an automatic geocoding system to solve the input toponyms into actual locations. We preprocess the inconsistencies before geocoding to increase the likelihood of having a resolvable toponym in the origin or destination fields.

Our approach for dealing with the inconsistencies before running the geocoder on the data was as follows. First, we checked if the toponyms had easily identifiable irregularities in the name, such as question marks or symbols indicating a character is out of range and replaced these toponyms of the city with the toponym of the institution. Second, we checked if the receiving or sending city were missing entirely, or if they were fully written in question marks, and we replaced it with the name of the institution. From the total of 2 282 950 individual records of mobility, these conditions affected 5136 records for sending toponyms and, 9825 records for receiving toponyms.

To geocode the toponyms efficiently, we combined the origin and destination toponyms into a list and removed duplicates. This left us with 48 281 unique toponyms to geocode. We then ran our first wave of geocoding with Photon on these data. We used the columns that indicate the sending/receiving countries, cities and institutions (Table 1) as inputs for geocoding.

#### Dealing with errors

Geocoding the unique place names (n = 48 281) initially returned 46 493 successfully geocoded locations. The remaining 2494 toponyms were not resolved (about 5% of the total unique place names). To geocode these place names, we needed to check the input toponyms manually, and correct the place names if possible. To do this, we first ordered the unsuccessfully geocoded locations in order of popularity using the counts of flows per location to ensure the most contributing toponyms to the flow network are addressed first. Then we manually corrected the place names to represent the correct location on the level of the city. We did this by reviewing information on the countries, cities, and institutions related to the mobility, to retrieve the correct origin or destination cities. For instance, instead of the name of a city, the mobility record could reference a postal code of a neighbourhood in the city as the “Sending city”, posing a challenge for the geocoder. However, checking which institution was the sending institution would reveal the city either directly (e.g., University of Barcelona) or after a brief online search for the institution.

Most often, the cause of unsuccessful geocoding was the spelling of the place name in a language not supported the geocoding system (e.g., Paryžius instead of Paris), question marks in place of umlaut characters (J?rvenp??, instead of Järvenpää), a NUTS 3 code instead of a city, a numerical ID code referring to an institution, or typographical errors. On top of these, the geocoding appeared to have difficulties with some Czech placenames with several diacritical and accent marks, such as “Třebíč”, which has also been identified by Šimbera *et al*.^47^, and might be caused by how such toponyms are stored in the gazetteer. For the NUTS 3 codes, we used the largest city in the NUTS 3 region as the toponym to ensure a geocoded location at the LAU level as well. For the institutional ID codes, we searched the city of the institution from Erasmus’ database of participant institutions. We did this for all toponyms that had two or more mobilities associated with them, which left roughly 800 toponyms with a singular associated movement.

When the place names were manually corrected, we ran the geocoding again, first with Photon, which resolved most of the 2494 manually corrected toponyms, and finally, with Nominatim, which resolved an additional 236 toponyms Photon did not resolve. In the end, we disregarded the 539 unsuccessfully geocoded toponyms that were connected to a singular movement. The final total for geocoded locations in the data is 47 742, covering 99% of the unique toponyms in the data.

### Spatial aggregation

To aggregate the geocoded point locations to their corresponding LAU and NUTS 3 regional units, we performed a nearest neighbour spatial join to assign regional codes to each location. The nearest neighbour spatial join assigns the regional codes to the geocoded toponym points by first checking if the point intersects with the polygon of the spatial unit. If the point intersected, it was assigned the LAU/NUTS 3 code corresponding to the region. If the point did not intersect with any regional polygon, we performed a nearest neighbour search within a five-kilometre radius, and assigned the regional code of the closest polygon within the radius. This was done to reduce errors caused by points that are located over water bodies, e.g., in the Greek or Finnish archipelagos. If there is no polygon within the search radius, the point is removed.

**Fig. 1:**
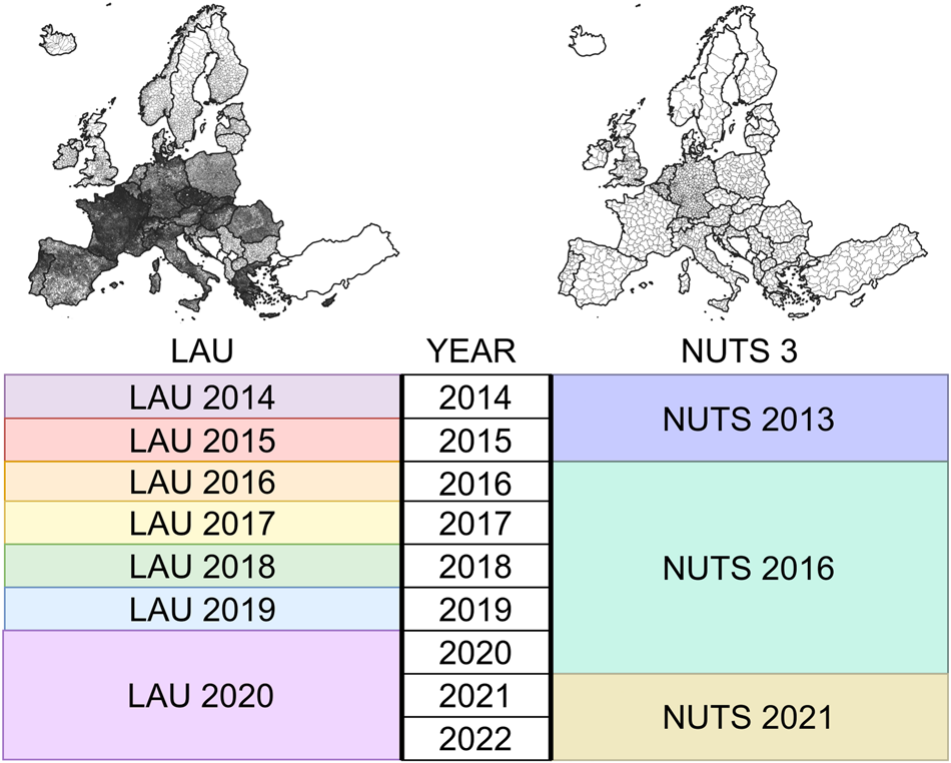
Temporal coverage of the NUTS and LAU spatial layers used in spatial aggregation for this data.

To cope with changes in the administrative structures on the LAU and NUTS 3 levels throughout the period, we used the corresponding calendar year’s LAU spatial layers and the most recent NUTS 3 layers in the generation of the OD matrices (see Fig. 1). For instance, for student mobility conducted in 2018, we used the LAU layer from 2018. To exemplify the aggregation to NUTS 3 level, we used the 2013 NUTS 3 version for mobility data from 2014 and 2015, and used the 2016 version of NUTS 3 for mobility data between 2016–2020, and finally the 2021 version for 2021–2022. This ensures interoperability with existing regional statistics from Eurostat or national statistical authorities, and is a common practice^28,37,49,50^. For the UK, we used the LAU layers from 2020 for mobility data covering 2021 and 2022 to maximise data coverage, as the UK LAU regions are not provided by Eurostat in the post-Brexit versions of the LAU layers.

As most of the locations in the data are at the level of cities, where educational institutions are located, we used LAU and NUTS 3 territorial divisions for spatial aggregation. These divisions are part of the standardised NUTS classification, which is a hierarchical system of nested territorial units used for official statistical data analyses and reporting in Europe, and in the European Union in particularly^51^. Because of the nested system, NUTS 3 is the most granular spatial level of the NUTS division, they can be easily converted to any other NUTS hierarchy level by dropping the trailing characters from NUTS 3 codes (see Section 5.5 for details). Therefore, aggregation to these spatial units ensures the widest use of this data by stakeholders beyond academia.

**Fig. 2:**
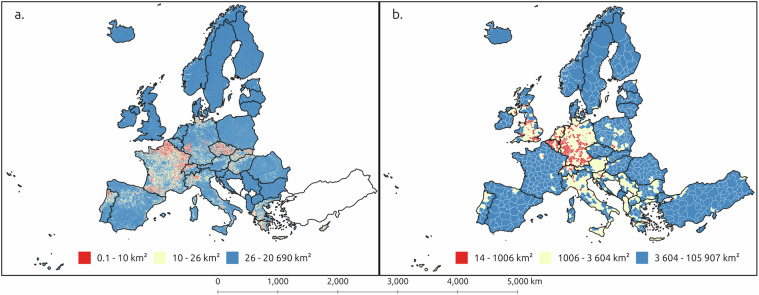
Map of LAU (**a**) and NUTS 3 (**b**) regions across continental Europe, visualised according to their geographical area using equal counts per class. LAU layers represent the 2020 version, while the NUTS 3 layers represent the 2021 version of the regional polygons. However, LAU regions do not include Turkey or Svalbard.

Generally, NUTS 3 regions contain regions that have a population in the range of 150 000 to 800 000 inhabitants, while the LAUs are sub-parts of NUTS 3 regions that roughly correspond to municipalities or other local administrative units. However, the geographical sizes of the areas in both datasets vary considerably (Fig. 2) from over 100 000 square kilometres to a few ten square kilometre areas for NUTS 3, and 20 000 square kilometres to a few hectares for LAU. Smaller regional unit sizes tend to be in Central Europe in both datasets, however there is some variation on where the smallest regions are located between the datasets. We have provided both levels of aggregation to enable a wider selection of analyses to be performed with this data.

After the aggregation, the geocoded data contains 47 742 locations that are within NUTS 3 and LAU polygons during 2014–2022. Using these locations to turn student mobility data into spatial data returns our final spatially enriched dataset containing 2 191 857 spatial movements across LAU/NUTS 3 regions within Europe. Of the student movements, 91 093 (4%) were filtered out as they did not have their origin and destination within Europe or had an unresolvable toponym. Therefore, the final dataset contains 96% of original data.

## Data Records

The final spatially enriched dataset is available from Väisänen *et al*.^52^ and contains 2 221 474 student movements between origins and destinations in total, representing interregional mobility flows of students aged 18 years old or older from 2014 until 2022. From these, 2 191 857 movements are at the NUTS 3 level, and 2 052 029 are at the LAU level. These movement flows capture the mobility of 2 275 868 individuals. Table 3 shows the annual numbers of all student movements and the total movements spatially assigned to both the LAU and NUTS 3 regional units in the individual-level data. The systematically larger data loss on LAU level is caused by the data not including LAU regions from Turkey and some other regions in Europe (e.g., Svalbard). The total number of participants taking part in the mobility is slightly higher than the number of movements in Table 3, as in some cases a single movement might have more than one participant. This happens when participants have an identical origin, destination, field of studies, gender, and duration of the mobility, as described by the Erasmus+ data dictionary provided by Directorate-General for Education, Youth, Sport and Culture^26^.

**Table 3:** Counts of movements in the spatially enriched individual mobility data within Europe at NUTS 3 and LAU spatial levels.

Year	Count of movements (NUTS3)	Count of movements (LAU)
2014	158 197	146 291
2015	261 600	242 574
2016	271 161	253 791
2017	279 695	262 659
2018	288 951	271 034
2019	288 237	270 373
2020	151 391	143 906
2021	235 508	219 957
2022	257 117	241 444
**Total**	2 191 857	2 052 029

LAU movements are fewer because the LAU data does not contain some regions the NUTS 3 data contains (see Fig. 2).

The spatially enriched dataset and the accompanying Python code with sufficient documentation for regenerating the data are available in a repository on Zenodo^52^. The repository also contains information on the requirements to run the scripts, the order to run the scripts in, and general information on the data release. The final dataset contains four tabular data files containing the spatially enriched mobility data:

- Erasmus_2014–2022_individual.csv - the full individual-level student mobility dataset
- Erasmus_2014–2022_individual.parquet.gzip - the full individual-level student mobility dataset in a compressed parquet file
- Erasmus_2014–2022_aggregate_LAU.csv - the aggregated student mobility flow dataset between LAU units
- Erasmus_2014–2022_aggregate_NUTS.csv - the aggregated student mobility flow dataset between NUTS 3 units

The individual-level mobility data contain all flows for students, who are at least 18 years old, and whose mobility has lasted at least 90 days. The individual-level data enables more granular analytical approaches, as it retains the original descriptive variables present in the initial Erasmus+ data. The origins and destinations are represented as regional codes both at LAU and NUTS 3 levels, but the city and institution names are present as well (see Table 4). As the CSV file of the individual data is approximately 1 GB in size, we have also provided a compressed parquet file of the same data for convenience. We include the CSV version as it is the most commonly used by stakeholders outside academia to maximise the use of the data.

**Table 4:** Variables in the spatially enriched individual-level data product.

Variable	Description	Value unit
Project reference	Erasmus+ project code	Text
Academic year	Year of the mobility	Text
Mobility start	Start of the mobility	Date
Mobility duration	Duration of mobility in calendar days	Integer
Activity	Type of mobility within Erasmus+ programme	Text
Field	Field name of the action	Text
Field of education	Field of education	Text
Participant country	Country of the participant	Text
Educational level	ISCED code	ISCED 3-9
Participant gender	Gender of the participant	Male/female/undefined
Participant profile	Describes whether participant is a learner or staff member	Learner/staff
Fewer opportunities	Describes whether participant comes from less privileged background	Yes/No
Participant age	Age of participant	Integer
Sending country	Country of origin for participant	Text
Sending city	City of origin for participant	Text
Sending organization	Institution of origin for participant	Text
Receiving country	Country of destination for participant	Text
Receiving city	City of destination for participant	Text
Receiving organization	Institution of destination for participant	Text
Actual participants	Number of participants with identical mobility	Integer
Origin	Origin toponym used in geocoding	Text
Destination	Destination toponym used in geocoding	Text
Year	Calendar year of mobility	Integer
orig_LAU	The GISCO LAU ID for the origin LAU	Text
dest_LAU	The GISCO LAU ID for the destination LAU	Text
orig_NUTS	The NUTS 3 code for the origin region	Text
dest_NUTS	The NUTS 3 code for the destination region	Text

The variables in the lower box represent the geocoding results added through this work.

The aggregated mobility flow data are in two CSV files in this data, one for flows using LAU units, and the other using NUTS 3 units. The file names indicate the spatial layers the flows are represented on. The flows use NUTS 3 and LAU unit versions corresponding to the year of the flows as per Fig. 1. The two aggregated files are simpler in the data structure compared to the individual data, and contain only seven variables (see descriptions from Table 5).

**Table 5:** Variables in the finalized aggregated data product.

Variable	Description	Value unit
OD_ID	Origin-destination id	Text
ORIGIN	LAU/NUTS 3 code of origin	Text
DESTINATION	LAU/NUTS 3 code of destination	Text
O_NAME	Name of origin LAU/NUTS 3 unit	Text
D_NAME	Name of destination LAU/NUTS 3 unit	Text
YEAR	Calendar year of mobility	Integer
COUNT	Count of participants	Integer

The spatially enriched dataset and the associated code for regenerating the data are available from Zenodo^52^ and licensed under Creative Commons Attribution 4.0. The code is also available from our GitHub repository at https://github.com/DigitalGeographyLab/mobitwin-erasmus.

## Technical Validation

To validate the accuracy of the geocoding at the NUTS 3 level, we selected a weighted random sample of 1000 locations from the total of 47 742 geocoded locations representing 2.1% of all possible locations in the data. We manually assessed whether the geocoded locations were in the correct NUTS 3 regions, similarly to de Rassenfosse *et al*.^37^. We used weighting in the sampling so that the locations that occur more frequently at either the origin or the destination have a higher likelihood of being included in the sample, and thus the validation we perform captures the reliability of the spatially enriched dataset better.

We focused our validation on the NUTS 3 regional level for three reasons. First, the LAU level does not contain Turkey and some other regions in Europe and thus would exclude student mobility to and from these regions from the data validation. Second, NUTS 3 regions are geographically larger than LAU, and thus our validation covers a larger proportion of the data as one NUTS 3 region will “collect” more incoming and outgoing movements compared to a singular LAU region. Finally, as the original data is largely at the city level and many of the larger cities in Europe are divided into numerous LAU units, validating on the NUTS 3 level makes more sense as NUTS 3 units cover full cities better.

We performed manual validation at the NUTS 3 level using the 2021 version of the spatial layer and flow data from 2021 and 2022 (see Fig. 1). We focused on one spatial layer because the output from geocoding a toponym is a geographic coordinate point expressed as latitudes and longitudes. Consequently, if the point is correct for one NUTS 3 version of the data, it is highly likely to be correct for the other NUTS 3 layer versions. To exemplify, the geocoded toponym of “Madrid, Spain” has the same geographic point coordinates regardless of the year, and if the coordinates are in Madrid, then it will be within the appropriate NUTS 3 and LAU regions across all the years.

Our manual validation shows the geocoding provides highly accurate results (Table 6). Out of the 1000 randomly sampled locations, 96.6% were geocoded to the correct NUTS 3 region. The accuracy increases to 99.4% when weighting for the occurrence of origins and destinations of movements in the dataset. This indicates that the incorrectly geocoded locations occur with student mobility between locations, which have a small number of students and their influence on the general mobility structure is marginal. Table 6 provides the users of the spatially enriched data and understanding on the robustness of the data, and no data records have been removed from the data. See Section 5 for more on considerations and limitations regarding this data.

**Table 6:** Results of manual validation of geocoding accuracy for origin-destination pairs and the number of movements they represent from 2021 and 2022.

	In correct NUTS3	Not in correct NUTS3	Total	Accuracy
**Sampled locations**	966	34	1000	96.6%
**Movement origins**	385 031	2 314	387 345	99.403%
**Movement destinations**	384 986	2 243	387 229	99.421%

## Usage Notes

This student mobility dataset with three data tables is readily available for a variety of applications for immediate use, requiring minimal processing. The aggregated and participant-level mobility data we provide enable several types of analysis, ranging from pan-European studies focusing on large mobility structures to more detailed examinations such as exploring the impact of incoming and outgoing students for local economies on the LAU level. The ready-made data set simplifies conducting a European analysis from a municipality to country level. The NUTS 3 level can easily be aggregated to NUTS 2, 1 and 0 (country) levels^51^. Moreover, it enables linking these data with any other official regional statistics provided by Eurostat. For more nuanced analyses, we recommend using the participant-level data, where the individual movements can be explored alongside the original descriptive variables provided by Erasmus. Finally, to support open science and to ensure transparency and customisability of our approach to more specific needs, the data production scripts are available online at Zenodo^52^ and at our GitHub https://github.com/DigitalGeographyLab/mobitwin-erasmus. Next, we provide some use cases based on the spatially enriched student mobility dataset.

There are some considerations to be aware of when using these data. First, the data cover only student mobility from the Erasmus+ programme between regions of the European Union and additional associated countries in Europe. Similarly, the data covers most of the student mobility within Europe, but not all movements, as there might also be bilateral exchange programmes and institution-specific scholarships that are not a part of the Erasmus+ programme. Second, the data might contain false positive locations, as seen in Section 4. For example, if a country has two cities with an identical name. In such cases, the Nominatim gazetteer, used by both Photon and Nominatim geocoders, favours the place which it considers to be more prominent through its inner ranking^48^. This could affect the LAU level data more than it does the NUTS 3 data, as the higher level NUTS 3 division has a higher likelihood to be correct. Third, our spatially enriched dataset contains mobilities of students who are at least 18 years old and whose mobility (i.e. student exchange) lasts for 90 days or longer. Therefore, the data excludes student mobilities related to short exchanges, research visits, and excursions of school children that are not linked to temporarily residing abroad and officially enrolling with the hosting institution. Fourth, in 2018, there were 178 missing names of LAU units in the aggregated data from Norway and Slovenia originating from the original LAU data provided by GISCO. The missing names do not affect the usability of the data, as the spatial units’ unique identifiers and geometries are correct.

### Regional brain gain and drain dynamics

**Fig. 3:**
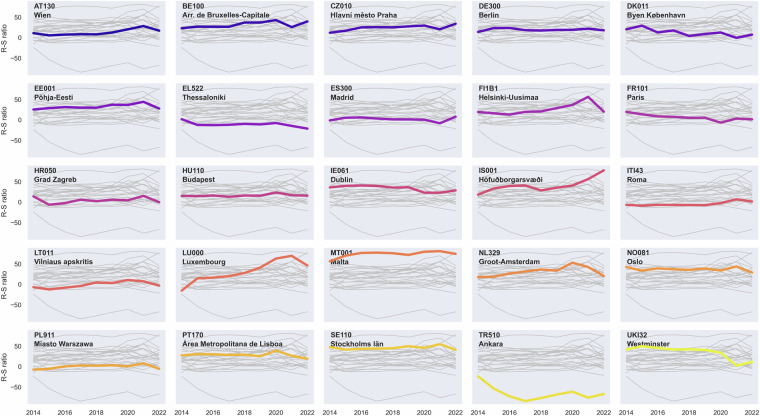
Receiving-sending ratios across several NUTS 3 regions across Europe. Positive values indicate the region is receiving more students, while negative values indicate the region is sending more students.

The Erasmus dataset can be used in analysing brain drain and brain gain dynamics at the regional level and how COVID-19 affected these dynamics. By examining the inflow and outflow of students in different regions, it is possible to identify areas that are successfully attracting academic talent and those experiencing a loss of skilled individuals (see Fig. 3). Understanding these trends enables regional policymakers to design targeted interventions to either retain or attract students and graduates. In regions experiencing a brain drain, the data can be instrumental in justifying investments in higher education initiatives and development projects aimed at retaining talent.

**Fig. 4:**
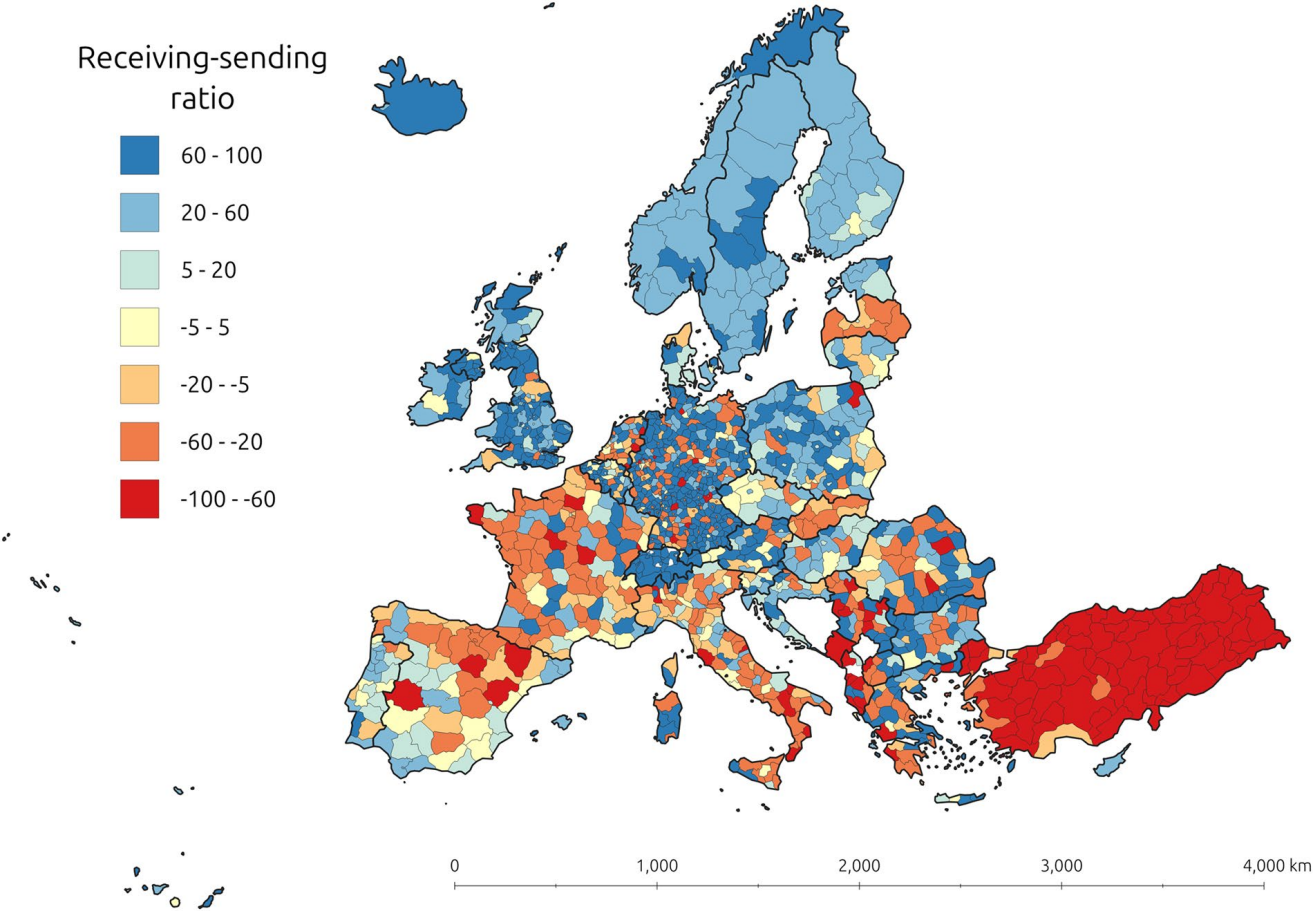
Ratios of between received and sent student movements across NUTS 3 regions and all years of the data using the standard NUTS 3 regions.

To exemplify this, Fig. 3 illustrates the variation in the stability of the ratio between received and sent students. It appears that regions with larger cities (e.g. Vienna, Berlin, Madrid), have a more stable ratio between received and sent students, whereas regions with smaller cities have more fluctuations (e.g., Malta and Helsinki-Uusimaa). COVID-19 also had a noticeable effect during 2020 in several regions, where the ratio reduced in many regions. After most countries had lifted COVID-19 restrictions, most regions have returned to pre-COVID ratio levels, although there are some exceptions. Figure 4 presents the geographical distribution of sending-to-receiving ratios across Europe, which also illustrates the regional side of brain gain and brain drain dynamics. Consequently, this dataset can provide valuable insights about the influence of COVID-19 on brain gain and brain drain dynamics in Europe.

### Academic cross-pollination and European identity

**Fig. 5:**
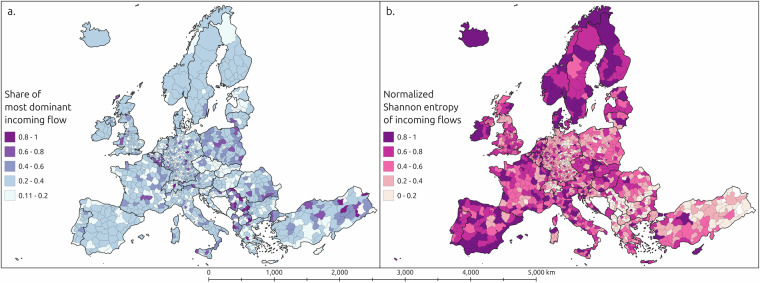
Maps of (**a**) the proportion of the most dominant country in incoming flows per NUTS 3 regions, and (**b**) the normalised Shannon entropy of the incoming flows’ countries. Higher values in the flow dominance map (**a**) indicate the incoming flows to the region are dominated by a single country to a greater extent. Higher values in the Shannon entropy map (**b**) indicate higher diversity of countries in the incoming flows to the region.

The goals of the Erasmus+ programme are to spread academic ideas and to support the formation of a shared European identity of EU citizens and citizens of partner and candidate states^22,24,25^. By exploring the diversity and dominance of incoming flows per region, we can examine regional academic cross-pollination, and which regions are the hot spots for diverse intercultural student interactions and thus play a crucial role in the formation of a European identity (see Fig. 5. These analyses can reveal which countries constitute the “cores” of academic cross-pollination and the European identity being formed through the programme, and which constitute the “periphery”. From such preliminary analyses, it is possible to continue examining whether the core-periphery structure could be contradictory to the goals of the European Union’s regional cohesion policies, which aim to reduce regional inequality^53^.

Figure 5 illustrates how diverse the flows of student mobility into NUTS 3 regions are based on the countries of origin. The diversity metrics used are Berger-Parker dominance (Fig. 5a) and normalised Shannon entropy (Fig. 5b), which are commonly used to assess diversity in various research contexts^54–56^. Berger-Parker dominance is a simple metric indicating how much the class with most observations dominates the sample, here it shows the proportion of the most dominant country of the incoming flows per region. Normalized Shannon entropy describes the diversity of the sample, scaled according to the theoretical maximum entropy of the sample if all observations were distributed equally across classes^54,55^. These analyses can reveal how narrow the student inflow is regarding their geographical scope. For example, some regions in Belgium, Poland and the Balkans seem to be more dependent on a singular origin of incoming students (Fig. 5).

These results can have implications for regional education policy, academic competitiveness, and resilience of international academic cooperation initiatives. In contrast, most regions in countries like Ireland, Spain, Portugal, Italy and much of Northern Europe seem to be hotspots for diverse student interaction, that is, they seem to be important regions for academic cross-pollination and innovation spread, but also for fostering the European identity through cross-cultural interactions.

In addition, the data set can be used for various network analyses of student mobility throughout the years, e.g. to map the changes in academic collaborations across Europe. Through network analyses, researchers can identify which regions act as central nodes within the European academic exchange network, and how the importance of nodes changes depending on origin node. These analyses can highlight existing collaboration patterns and identify opportunities to strengthen partnerships between institutions, thereby enhancing the overall academic network across the continent. Conversely, the data may reveal whether mobility programs help bridge educational disparities by providing increased access to international opportunities for students from less advantaged areas.

### Role of student mobility for regional economies

**Fig. 6:**
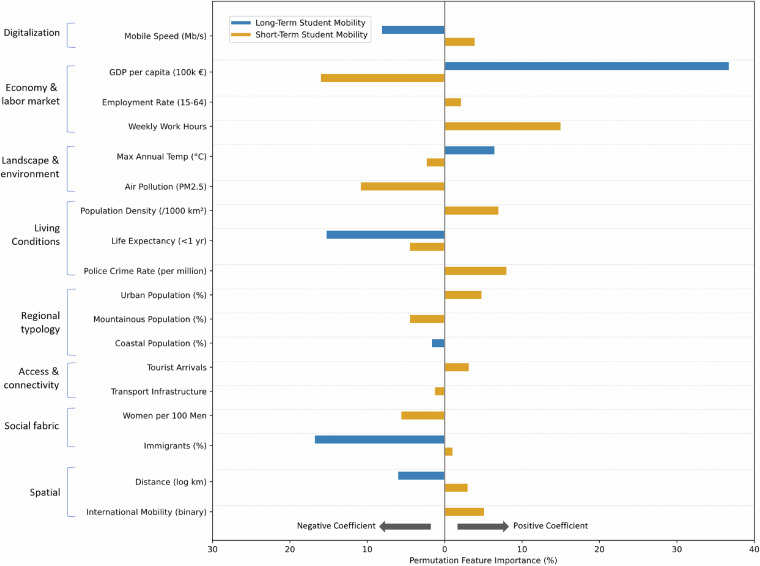
Influence of regional characteristics on short- and long-term student mobility on the NUTS 2 level. Permutation feature importance indicates how important the characteristic is for student mobility, whereas the direction indicates the influence is positive or negative.

The data can also be used in evaluating the economic effects of student mobility on regional economies. By integrating this dataset with other regional characteristics, for example from Eurostat, it becomes possible to assess how student mobility influences economic activities such as the tourism, housing, and retail sectors. Figure 6 shows which regional characteristics play a role in explaining short-term (3–11 months) and long-term (longer than 11 months) student mobility on the NUTS 2 level.

More nuanced analysis using a multi-level approach can be obtained by using individual level data that can be combined with local area and regional characteristics. These analyses can provide a quantifiable understanding of the contribution of student mobility to regional economic growth. Furthermore, the dataset can inform policymakers working with urban planning and infrastructure investment, as local governments can gain valuable insights into the economic benefits of attracting international students.

### Visualisation of complex flow data

**Fig. 7:**
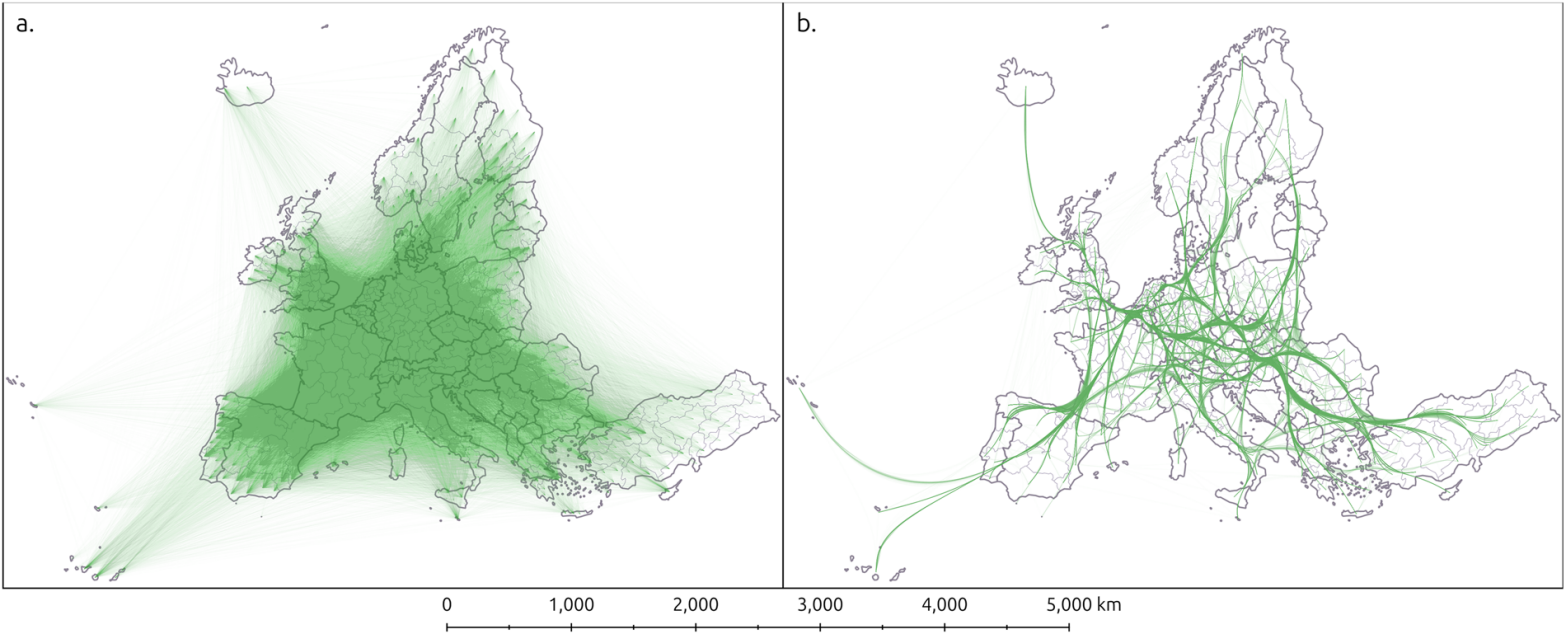
Maps of flows as (**a**). straight line geometries on NUTS 3 level and (**b**) after edge-path bundling on NUTS 2 level. This visualisation uses similar blending modes and transparency settings for the line geometries for both maps to illustrate the effect of edge bundling.

One powerful way of disseminating findings from human mobility research is through geographic visualisations. However, cartographic representations of student mobility, that has thousands of flows between hundreds of spatial units, is a challenging task. Conventionally, one would visualise the flows by connecting the origin and destination points with a straight line, often with an arrow to indicate direction. With large-scale flow data, this type of visualisation easily becomes a “fuzzy mess” of criss-crossing lines. This makes visual interpretations and dissemination of findings difficult (see Fig. 7a).

To overcome this visualisation issue, we suggest using edge bundling to simplify the line geometries of the mobility data. Edge bundling is a family of visualisation techniques whereby edges that are similar in direction and proximity are “bundled” together to reduce visual clutter in massive flow data^57^. By using the edge bundling tool developed by Väisänen *et al*.^58^, visualisation of complex flow data, such as Erasmus+ student mobility, is more feasible. The comparison between straight line geometries and edge-bundled geometries of the student flows clearly illustrates the difference (Fig. 7). By using our edge-bundling tool, it is possible to visualise student mobility flows linked to a certain country and thus make visual comparisons between countries and/or between years, for example.

### Converting mobility data to other NUTS levels

Given the hierarchical system of NUTS which contains nested territorial units at various NUTS divisional levels, the conversion of student mobility at the NUTS 3 level to any other higher NUTS level is simple and straightforward. The nested hierarchy is included in the identifiers of the NUTS spatial units (i.e. NUTS ID codes) as a higher-level spatial unit code is nested into a lower-level spatial unit code. For example, the NUTS 3 spatial unit code “ES511” (Barcelona, Spain) can be converted to a NUTS 2 code by excluding the trailing character “ES51”, which refers to Catalonia, the higher-level NUTS 2 spatial unit containing Barcelona and other NUTS 3 regions that are within Catalonia. Further, our NUTS 3 level data can be aggregated to a country level (NUTS 0) by selecting the first two characters representing a country - “ES” refers to Spain. After dropping the trailing character, performing a simple grouping operation by the new NUTS ID code will correctly aggregate the data into the desired NUTS level.

**Table 7:** Table describing author contributions.

Author	Data curation	Analysis	Writing - original draft	Writing - review & editing
Tuomas Väisänen	X	X	X	X
Milad Malekzadeh	—	X	—	X
Oula Inkeröinen	X	—	—	—
Olle Järv	—	X	—	X

## Data Availability

The Python code used to produce this data set is available from the Zenodo repository associated with this article^52^, but also from our GitHub repository at the following link: https://github.com/DigitalGeographyLab/mobitwin-erasmus.

## References

[1] Kaufmann, V. Mobility as a Tool for Sociology. *Sociologica*10.2383/77046 (2014).

[2] Järv, O., Aagesen, H. W., Väisänen, T. & Massinen, S. Revealing mobilities of people to understand cross-border regions: Insights from Luxembourg using social media data. *European Planning Studies* **31**, 1754–1775, 10.1080/09654313.2022.2108312 (2023).

[3] Schmahmann, L., Poorthuis, A. & Chapple, K. Pandemic polycentricity? Mobility and migration patterns across New York over the course of the Covid-19 pandemic. *Cambridge Journal of Regions, Economy and Society* **15**, 515–535, 10.1093/cjres/rsac017 (2022).

[4] Müürisepp, K., Järv, O., Sjöblom, F., Toger, M. & Östh, J. Segregation and the pandemic: The dynamics of daytime social diversity during COVID-19 in Greater Stockholm. *Applied Geography* **154**, 10.1016/j.apgeog.2023.102926 (2023). 10.1016/j.apgeog.2023.102926 PMC9998301 36999002

[5] Rowe, F., Calafiore, A., Arribas-Bel, D., Samardzhiev, K. & Fleischmann, M. Urban exodus? Understanding human mobility in Britain during the COVID-19 pandemic using Meta-Facebook data. *Population, Space and Place* **29**, e2637, 10.1002/psp.2637 (2023). 36718419 10.1002/psp.2637 PMC9877951

[6] Steenbruggen, J., Tranos, E. & Nijkamp, P. Data from mobile phone operators: A tool for smarter cities? *Telecommunications Policy* **39**, 335–346, 10.1016/j.telpol.2014.04.001 (2015).

[7] Gérard, M. & Sanna, A. Students’ mobility at a glance: efficiency and fairness when brain drain and brain gain are at stake. *Journal of international Mobility* **5**, 43–74, 10.3917/jim.005.0043 (2017).

[8] Breznik, K. & Skrbinjek, V. Erasmus student mobility flows. *European Journal of Education* **55**, 105–117, 10.1111/ejed.12379 (2019).

[9] Cuzzocrea, V. & Krzaklewska, E. Erasmus students’ motivations in motion: Understanding super-mobility in higher education. *Higher Education* **85**, 571–585, 10.1007/s10734-022-00852-6 (2023). 35496498 10.1007/s10734-022-00852-6 PMC9034258

[10] Earls, C. W. Popping the Erasmus Bubble: Perceptions of Intercultural Awareness and Competence of Incoming Erasmus+ Students and the Preparation Challenge. *Higher Education Research* **3**, 45–54, 10.11648/j.her.20180303.13 (2018).

[11] Nada, C. I. & Legutko, J. “Maybe we did not learn that much academically, but we learn more from experience” – Erasmus mobility and its potential for transformative learning. *International Journal of Intercultural Relations* **87**, 183–192, 10.1016/j.ijintrel.2022.03.002 (2022).

[12] Lesjak, M., Juvan, E., Ineson, E. M., Yap, M. H. T. & Axelsson, E. P. Erasmus student motivation: Why and where to go? *Higher Education* **70**, 845–865, 10.1007/s10734-015-9871-0 (2015).

[13] Rodríguez González, C., Bustillo Mesanza, R. & Mariel, P. The determinants of international student mobility flows: An empirical study on the Erasmus programme. *Higher Education* **62**, 413–430 (2011).

[14] Malet Calvo, D., Nofre, J. & Fuarros, Í. S. Unveiling the intersections between tourism industry and student mobility. *Population, Space and Place* **30**, e2752, 10.1002/psp.2752 (2024).

[15] Gadár, L., Kosztyán, Z. T., Telcs, A. & Abonyi, J. Cooperation patterns in the ERASMUS student exchange network: An empirical study. *Applied Network Science* **7**, 74, 10.1007/s41109-022-00512-9 (2022).

[16] Giousmpasoglou, C. & Koniordos, S. K. Brain drain in higher education in Europe: Current trends and future perspectives. In Giousmpasoglou, C., Marinakou, E. & Paliktzoglou, V. (eds.) *Brain Drain in Higher Education: The Case of the Southern European Countries and Ireland*, 229–262, http://www.novapublishers.com/ (Nova Science Publishers, New York, 2017).

[17] Rasamoelison, J. D., Averett, S. & Stifel, D. International student-migrant flows and growth in low- and middle-income countries: Brain gain or brain drain? *Applied Economics* **53**, 3913–3930, 10.1080/00036846.2021.1886237 (2021).

[18] Wende, M. V. D. International Academic Mobility: Towards a Concentration of the Minds in Europe. *European Review* **23**, S70–S88, 10.1017/S1062798714000799 (2015).

[19] Faggian, A., Corcoran, J. & Rowe, F. Special issue on youth and graduate migration. *The Annals of Regional Science* **59**, 571–575, 10.1007/s00168-017-0845-2 (2017).

[20] Labrianidis, L., Sykas, T., Sachini, E. & Karampekios, N. Socioeconomic status of international students and its relation to the brain drain: Evidence from Greek PhD holders. *International Journal of Social Economics* **51**, 757–770, 10.1108/IJSE-09-2022-0607 (2023).

[21] Directorate-General for Education, Youth, Sport and Culture. *Erasmus+ Annual Report 2023*10.2766/833629 (Publications Office of the European Union, Luxembourg, 2024).

[22] Oborune, K. Becoming more European after ERASMUS? The Impact of the ERASMUS Programme on Political and Cultural Identity. *Epiphany. Journal of Transdisciplinary Studies* **6**, 182–202, 10.21533/epiphany.v6i1.60 (2013).

[23] Croce, G. & Ghignoni, E. The Multifaceted Impact of Erasmus Programme on the School-to-Work Transition: A Matching Sensitivity Analysis. *Research in Higher Education* **65**, 732–754, 10.1007/s11162-024-09774-x (2024).

[24] European Commission. The UK and Erasmus+ https://erasmus-plus.ec.europa.eu/the-uk-and-erasmus (2024).

[25] European Union. The Erasmus programme moves ahead - A first series of community aids to 240 inter-university cooperation programmes https://ec.europa.eu/commission/presscorner/detail/en/ip_87_423 (1987).

[26] Directorate-General for Education, Youth, Sport and Culture. Erasmus+ Mobility Raw Data https://data.europa.eu/data/datasets/erasmus-mobility-raw-data?locale=en (2024).

[27] Komoot. Komoot/Photon. https://github.com/komoot/photon (2024).

[28] Gadár, L., Kosztyán, Z. T., Telcs, A. & Abonyi, J. A multilayer and spatial description of the Erasmus mobility network. *Scientific Data* **7**, 41, 10.1038/s41597-020-0382-1 (2020). 32029724 10.1038/s41597-020-0382-1 PMC7005280

[29] Eurostat. Copyright notice - Documentation of data.europa.eu (DEU) https://dataeuropa.gitlab.io/data-provider-manual/legal-notice/copyright/ (2025).

[30] European Commission. 2011/833/EU: Commission Decision of 12 December 2011 on the reuse of Commission documents https://eur-lex.europa.eu/eli/dec/2011/833/oj/eng (2011).

[31] UNESCO. International Standard Classification of Education ISCED 2011. Tech. Rep. UIS/2012/INS/10/REV, UNESCO Institute of Statistics, Montreal, Canada https://uis.unesco.org/sites/default/files/documents/international-standard-classification-of-education-isced-2011-en.pdf (2012).

[32] European Commission, European Education and Culture Executive Agency, Ferencz, I. & Kupriyanova, V.*20 Years of Erasmus Mundus: Beyond Borders and Boundaries*10.2797/767054 (Publications Office of the European Union, Luxembourg, 2024).

[33] Finnish National Agency for Education. Countries participating in the Erasmus+ programme https://www.oph.fi/en/programmes/countries-participating-erasmus-programme (2024).

[34] Leppämäki, T., Toivonen, T. & Hiippala, T. Geographical and linguistic perspectives on developing geoparsers with generic resources. *International Journal of Geographical Information Science* **38**, 2039–2060, 10.1080/13658816.2024.2369539 (2024).

[35] Pérez, V. & Aybar, C. Challenges in Geocoding: An Analysis of R Packages and Web Scraping Approaches. *ISPRS International Journal of Geo-Information* **13**, 170, 10.3390/ijgi13060170 (2024).

[36] Nguyen, H. L., Tsolak, D., Karmann, A., Knauff, S. & Kühne, S. Efficient and Reliable Geocoding of German Twitter Data to Enable Spatial Data Linkage to Official Statistics and Other Data Sources. *Frontiers in Sociology* **7**, 10.3389/fsoc.2022.910111 (2022). 10.3389/fsoc.2022.910111 PMC9220088 35755485

[37] de Rassenfosse, G., Kozak, J. & Seliger, F. Geocoding of worldwide patent data. *Scientific Data* **6**, 260, 10.1038/s41597-019-0264-6 (2019). 31695047 10.1038/s41597-019-0264-6 PMC6834584

[38] Hu, X., Kersten, J., Klan, F. & Farzana, S. M. Toponym resolution leveraging lightweight and open-source large language models and geo-knowledge. *International Journal of Geographical Information Science* **0**, 1–28, 10.1080/13658816.2024.2405182 (2024).

[39] Zhang, Z. & Bethard, S. A survey on geocoding: Algorithms and datasets for toponym resolution. *Language Resources and Evaluation*10.1007/s10579-024-09730-2 (2024).

[40] geopy contributors. Geopy. geopy https://github.com/geopy/geopy (2024).

[41] Nelson, T., Goodchild, M. & Wright, D. Accelerating ethics, empathy, and equity in geographic information science. *Proceedings of the National Academy of Sciences* **119**, 10.1073/pnas.2119967119 (2022). 10.1073/pnas.2119967119 PMC9171629 35507875

[42] Holbrook, J. B. Open Science, Open Access, and the Democratization of Knowledge. *Issues in Science and Technology* **35**, 26–28 (2019).

[43] OpenStreetMap. Nominatim. osm-search https://github.com/osm-search/Nominatim (2024).

[44] Clemens, K. Qualitative Comparison of Geocoding Systems using OpenStreetMap Data. *International Journal on Advances in Software* **8**, 377–386 (2015).

[45] Cetl, V., Kliment, T. & Jogun, T. A comparison of address geocoding techniques – case study of the city of Zagreb, Croatia. *Survey Review* **50**, 97–106, 10.1080/00396265.2016.1252517 (2018).

[46] Serere, H. N., Resch, B., Havas, C. R. & Petutschnig, A. Extracting and Geocoding Locations in Social Media Posts: A Comparative Analysis. *GI_Forum* **1**, 167–173, 10.1553/giscience2021_02_s167 (2021).

[47] Šimbera, J., Drbohlav, D. & Štych, P. Geocoding Freeform Placenames: An Example of Deciphering the Czech National Immigration Database. *ISPRS International Journal of Geo-Information* **10**, 335, 10.3390/ijgi10050335 (2021).

[48] Nominatim. Place Ranking https://nominatim.org/release-docs/latest/customize/Ranking/ (2025).

[49] Rosvold, E. L. & Buhaug, H. GDIS, a global dataset of geocoded disaster locations. *Scientific Data* **8**, 61, 10.1038/s41597-021-00846-6 (2021). 33594086 10.1038/s41597-021-00846-6 PMC7887188

[50] Macdonald, J. L., Dolega, L. & Singleton, A. An open source delineation and hierarchical classification of UK retail agglomerations. *Scientific Data* **9**, 541, 10.1038/s41597-022-01556-3 (2022). 36057644 10.1038/s41597-022-01556-3 PMC9440905

[51] European Commission & Eurostat. *Regions in the European Union Nomenclature of Territorial Units for Statistics (NUTS) – 2024 Edition*10.2785/714519 (Publications Office of the European Union, Luxembourg, 2024).

[52] Väisänen, T., Malekzadeh, M., Inkeröinen, O. & Järv, O. Mobility of Erasmus+ students in Europe: Geolocated individual and aggregate mobility flows from 2014 to 2022 10.5281/zenodo.14332354 (2025).10.1038/s41597-025-04789-040128262

[53] Treaty on the Functioning of the European Union. Consolidated version of the Treaty on the Functioning of the European Union - Part Three: Union Policies and Internal Actions - Title XVIII: Economic, Social and Territorial Cohesion - Article 174 (ex Article 158 TEC), Official Journal C115, European Union http://data.europa.eu/eli/treaty/tfeu_2008/art_174/oj/eng (2008).

[54] Holloway, S. R., Wright, R. & Ellis, M. The Racially Fragmented City? Neighborhood Racial Segregation and Diversity Jointly Considered. *The Professional Geographer* **64**, 63–82, 10.1080/00330124.2011.585080 (2012).

[55] Hiippala, T., Väisänen, T., Toivonen, T. & Järv, O. Mapping the languages of Twitter in Finland: Richness and diversity in space and time. *Neuphilologische Mitteilungen* **121**, 12–44, 10.51814/nm.99996 (2020).

[56] Väisänen, T., Järv, O., Toivonen, T. & Hiippala, T. Capturing urban diversity through languages: Long-term Changes in Multilingual Residential Neighbourhoods in the Helsinki Metropolitan Area. *Population, Space and Place* **30**, 10.1002/psp.2717 (2023).

[57] Wallinger, M., Archambault, D., Auber, D., Nöllenburg, M. & Peltonen, J. Edge-Path Bundling: A Less Ambiguous Edge Bundling Approach. *IEEE Transactions on Visualization and Computer Graphics* **28**, 313–323, 10.1109/TVCG.2021.3114795 (2022). 34587038 10.1109/TVCG.2021.3114795

[58] Väisänen, T., Inkeröinen, O., Malekzadeh, M. & Järv, O. Edge-bundling tool for regional mobility flow data. *Zenodo*10.5281/zenodo.14532548 (2024).

